# CSF Amino Acids, Pterins and Mechanism of the Ketogenic Diet

**DOI:** 10.15844/pedneurbriefs-29-10-6

**Published:** 2015-10

**Authors:** J. Gordon Millichap

**Affiliations:** 1 Division of Neurology, Ann & Robert H. Lurie Children's Hospital of Chicago, Chicago, IL; 2 Departments of Pediatrics and Neurology, Northwestern University Feinberg School of Medicine, Chicago, IL

**Keywords:** Ketogenic Diet, Neurotransmitters, Pterins, Amino Acids, Refractory Epilepsy

## Abstract

Investigators from Hospital Sant Joan de Deu, Barcelona, Spain, studied the relationship between the etiology of refractory childhood epilepsy, CSF neurotransmitters, pterins, and amino acids, and response to a ketogenic diet in 60 patients with refractory epilepsy, 83% focal and 52% idiopathic.

Investigators from Hospital Sant Joan de Deu, Barcelona, Spain, studied the relationship between the etiology of refractory childhood epilepsy, CSF neurotransmitters, pterins, and amino acids, and response to a ketogenic diet in 60 patients with refractory epilepsy, 83% focal and 52% idiopathic. Patients with GLUT-1 deficiency, pyruvate dehydrogenase deficiency, or other inborn error of metabolism were excluded. Mean age at epilepsy onset was 24 months. A 4:1 ratio ketogenic diet was followed in 40 patients, 3:1 ratio in 12, and 1 or 2:1 ratio in the remainder. The ketogenic diet was effective (> 50% reduction in seizure frequency) in 31.6% of patients, at 6 months after initiating the diet. Lysine and arginine CSF values analyzed in ketogenic diet responders were significantly lower than for nonresponders (P<0.05), but the remainder of a battery of amino acids and the glucose and lactic acid levels analyzed showed no differences for responders and nonresponders.. The rate of efficacy of the diet was not related to the etiologies of epilepsy nor to CSF pterins or biogenic amine concentrations. The authors consider that changes in biogenic amines and amino acids in CSF should be considered as potential mechanisms for the ketogenic diet efficacy in treatment of refractory epilepsy. [1]

COMMENTARY. The ketogenic diet was first introduced in the treatment of epilepsy in 1921, by Wilder [2] at the Mayo Clinic. He attributed the effect of the diet to ketosis and specifically to acetoacetic acid. Several theories for the anticonvulsant effect followed, many supported by effects in laboratory animals with experimental seizures and by clinical studies, and summarized in an editorial commentary in Epilepsia by Nordli and De Vivo in 1997 [3]. In earlier studies, the emphasis was on the effect of the diet on water and electrolyte balance, the anticonvulsant action correlated with an increased urinary excretion and negative balance of sodium and potassium, independent of acidosis and ketosis, and similar to the effect of acetazolamide [4]. De Vivo and associates (1978) reported changes in cerebral metabolites in chronically ketotic rats, but in contrast to clinical systemic studies, no alterations in brain water content, electrolytes, or pH [3]. Millichap and associates in balance studies (1964) in children with absence epilepsy found decreases in blood pH, PCO2, and standard bicarbonate [4]. Urinary excretion of electrolytes was increased, and the balance of sodium, potassium and other electrolytes was negative. The excretion of free amino acids was variable. Increase in level of leucine in the serum was the only change noted in amino acids. Fluid intake and urine output were reduced, and fall in body weight was initially rapid. Further studies comparing metabolic changes in ketogenic diet responders and nonresponders and significance of amino acid and pterin variations are indicated. Recent articles regarding the mechanism of action of the diet (previously reviewed in *Pediatric Neurology Briefs*) reemphasize the importance of ketone bodies as a factor [5], and introduce a novel mechanism and potential treatment with LDH inhibitors [6].
